# Rats that differentially respond to cocaine differ in their dopaminergic storage capacity of the nucleus accumbens

**DOI:** 10.1111/j.1471-4159.2008.05323.x

**Published:** 2008-06

**Authors:** Michel M M Verheij, Eric L W de Mulder, Elvira De Leonibus, Karen M J van Loo, Alexander R Cools

**Affiliations:** * Department of Cognitive Neuroscience, Division of Psychoneuropharmacology, Radboud University of NijmegenNijmegen, The Netherlands; † Department of Genetics and Molecular Biology, La Sapienza University of RomeRome, Italy; ‡ Telethon Institute of Genetics and MedicineNaples, Italy; § Department of Molecular Animal Physiology, Nijmegen Center for Molecular Life Sciences, Radboud University of NijmegenNijmegen, The Netherlands

**Keywords:** cocaine pharmacology, dopamine pools, individual differences, plasmalemmal transporters, reserpine pharmacology, vesicular transporters

## Abstract

Cocaine (COC) inhibits the re-uptake of dopamine. However, the dopamine response to COC also depends on dopamine inside storage vesicles. The aim of this study was to investigate whether rats that differentially respond to COC differ in their dopaminergic storage capacity of the nucleus accumbens. Total and vesicular levels of accumbal dopamine as well as accumbal vesicular monoamine transporter-2 levels were established in high (HR) and low responders (LR) to novelty rats. Moreover, the effects of reserpine (RES) on the COC-induced increase of extracellular accumbal dopamine were investigated. HR displayed higher accumbal levels of total and vesicular dopamine than LR. Moreover, HR displayed more accumbal vesicular monoamine transporters-2 than LR. COC increased extracellular accumbal dopamine more strongly in HR than in LR. A low dose of RES prevented the COC-induced increase of accumbal dopamine in LR, but not in HR. A higher dose of RES was required to inhibit the COC-induced increase of accumbal dopamine in HR. These data demonstrate that HR were marked by a larger accumbal dopaminergic storage pool than LR. It is hypothesized that HR are more sensitive to COC than LR, because COC can release more dopamine from accumbal storage vesicles in HR than in LR.

*J. Neurochem.* (2008) **105,** 2122–2133.

Individual differences in the susceptibility to psychostimulants have extensively been reported, both in humans (Jaffe and Archer 1987; Ball *et al.* 1994; Gynther *et al.* 1995; van den Bree *et al.* 1998) and in animals (Piazza *et al.* 1989, 2000; Mantsch *et al.* 2001). This study focused on two types of rat that differ in their acute response to cocaine (COC). These individuals are selected on the basis of their locomotor response to a novel open-field and, accordingly, labeled high (HR) and low responders (LR) to novelty (Piazza *et al.* 1989, 1991b; Rouge-Pont *et al.* 1993; Dellu *et al.* 1996; Bevins *et al.* 1997; Cools and Gingras 1998; Cools and Tuinstra 2003; Kabbaj 2004). Previous studies have demonstrated that COC increases the locomotor response and the extracellular levels of accumbal dopamine more strongly in HR than in LR (Hooks *et al.* 1991b; Chefer *et al.* 2003).

Cocaine inhibits the re-uptake of monoamines by blocking plasmalemmal monoamine transporters (Lee *et al.* 2001). Several studies have suggested that individual differences in the re-uptake of dopamine may explain individual differences in the response to COC (Sabeti *et al.* 2002, 2003; Chefer *et al.* 2003; Briegleb *et al.* 2004; Zahniser and Sorkin 2004). However, behavioral and neurochemical studies have demonstrated that the response to COC depends on storage vesicles as well (Scheel-Kruger *et al.* 1977; McMillen *et al.* 1980; McMillen 1983; Davis 1985; Hurd and Ungerstedt 1989; Sulzer and Rayport 1990; Florin *et al.* 1995; Pifl *et al.* 1995; Venton *et al.* 2006). It is unknown to what extent individual differences in the dopaminergic storage capacity contribute to individual differences in response to COC. The above-mentioned finding that HR are marked by a larger COC-induced increase of accumbal dopamine than LR suggests that HR store more accumbal dopamine inside storage vesicles than LR. Accordingly, total and vesicular levels of accumbal dopamine were measured in both types of rat. Given that vesicular monoamine transporters-2 (VMAT-2) control the amount of dopamine inside storage vesicles (Pothos *et al.* 2000; Pothos 2002), the levels of the accumbal VMAT-2 were also measured. Based on the notion that LR store less accumbal dopamine inside vesicles than HR, it was hypothesized that the nucleus accumbens of LR contains less VMAT than the nucleus accumbens of HR.

The drug reserpine (RES) inhibits the VMAT-mediated uptake of cytoplasmatic monoamines into storage vesicles (Kirshner *et al.* 1963; Henry *et al.* 1998). As the extracellular levels of monoamines strongly depend on an intact shuttle between cytoplasmatic and vesicular monoamines (Schoemaker and Nickolson 1983; Leviel *et al.* 1989; Arbuthnott *et al.* 1990), RES decreases the extracellular levels of accumbal dopamine (Verheij and Cools 2007). The present study also investigated the effects of RES on the COC-induced increase of extracellular accumbal dopamine. It was hypothesized that COC-treated LR, which are supposed to be marked by a relatively small storage pool containing low amounts of VMAT, are more vulnerable to the RES-induced dopamine depletion than COC-treated HR, which are supposed to be marked by a relatively large storage pool containing high amounts of VMAT.

## Experimental procedures

See Appendix S1 for the full version of this section.

### Open-field selection

The open-field selection is described by Cools *et al.* (1990). Rats that habituated in less than 480 s and walked less than 4800 cm in 30 min were labeled LR. Rats that habituated after 840 s and walked more than 6000 cm in 30 min were labeled HR; 48 adult male LR and 59 adult male HR that were selected from the outbred strain of Nijmegen Wistar rats were used throughout this study.

### Vesicular levels of accumbal dopamine (experiment 1)

**Fig. 1:**
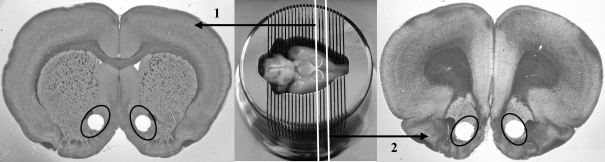
Representative placement of accumbal punches. The brain was placed in a brain matrix. The first incision was made at the position where the two optic nerves fuse with the optic chiasm. The second incision was made 3 mm rostral to this point. Coronal incisions were similar to anterior/posterior 9.0 and 12.0 mm of Paxinos and Watson (1986). The anterior commissure was used as a landmark to reliable punch out accumbal tissue. For each rat, tissue of the left and right punch was pooled. Correctly placed punches were located within the area of the black oval.

The aim of this experiment was to measure the levels of vesicular dopamine in punches of the nucleus accumbens. Seven days after the open-field selection, 10 LR and 10 HR were killed by decapitation. Their brains were quickly removed, immediately placed into a brain matrix, and subsequently sectioned at 3.0 mm intervals (Mong *et al.* 2003). Coronal incisions were made at anterior/posterior 9.0 and 12.0 mm (Paxinos and Watson 1986). The most caudal incision was made at the position where the two optic nerves fuse with the optic chiasm (Fig. 1). From the identified slice, one punch of accumbal tissue was obtained from either side of the brain using a 1.22 mm i.d. stainless steel needle (Szczypka *et al.* 2001). The anterior commissure was used as a landmark to reliable punch out accumbal tissue (Fig. 1). For each rat, tissue of the left and right punch was pooled (total volume 2 × 3.5 mm^3^ = 7.0 mm^3^). The remaining tissue was fixated in *p*-formaldehyde solution in order to allow histological verification of the exact position of the punch needle. Purified accumbal vesicles were prepared by ultracentrifugation (Staal *et al.* 2000). Vesicular dopamine levels were obtained according to the procedures described by Sandoval *et al.* (2003). The final supernatant was injected into a high performance liquid chromatography–electrochemical detection system for separation and quantification of vesicular dopamine. Vesicular dopamine levels were normalized for variation in protein loading using the total protein concentration of the first supernatant (Sandoval *et al.* 2003).

### Accumbal VMAT-2 levels and total levels of accumbal dopamine (experiment 2)

To investigate whether the putative individual differences in accumbal dopaminergic storage capacity were associated with individual differences in VMAT-2 levels, accumbal punches of 12 LR and 12 HR (isolated as described above) were analyzed for VMAT-2 expression. The punches were homogenized in phosphate-buffered saline containing urea, sodium dodecyl sulfate, β-mercaptoethanol, phenylmethylsulfonyl fluoride, and soybean trypsin inhibitor (Jensen *et al.* 1998). Next, proteins were separated on an 8% sodium dodecyl sulfate–polyacrylamide gel and transferred to nitrocellulose membranes. VMAT-2 expression was examined using the anti-VMAT-2 antibody (1 : 1000, AB1767; Chemicon, Hampshire, UK) and anti-β-tubulin antibody (1 : 3000, E7; Chu and Klymkowsky 1989). Band intensities were corrected for background.

For quantification of the total levels of accumbal dopamine, the samples that were used to asses the amount of VMAT were diluted and immediately injected into the high performance liquid chromatography–electrochemical detection system. VMAT and total dopamine levels were normalized for variation in protein loading using the levels of tubulin (Hedtjarn *et al.* 2002).

### Effects of reserpine on the cocaine-induced increase of extracellular accumbal dopamine (experiment 3)

The aim of this experiment was to investigate the effects of RES on the COC-induced increase of extracellular accumbal dopamine. A group of 26 LR and 37 HR were unilaterally implanted with a guide cannula directed at the right nucleus accumbens according to previously described procedures (Verheij and Cools 2007). The rats were allowed to recover from surgery for the next 7–10 days in dialysis cages. At the first day of the experiment, a dialysis probe was inserted into the guide cannula. Four hours following probe insertion, HR and LR were injected with RES or its solvent (see below). At the second day of the experiment, accumbal dialysates were analyzed for dopamine according to previously described procedures (De Leonibus *et al.* 2006). As soon as the dopamine samples differed less than 10%, three baseline samples were taken. The average of these three samples served as control value (100%) to study the drug-induced changes of accumbal dopamine. Immediately after the third baseline sample was taken, rats that were treated with RES or its solvent on day 1 were injected with COC or saline (see below). These rats were subsequently exposed to a cage that was slightly larger than their home cage and lacked sawdust on the floor (Verheij and Cools 2007). After this exposure to novelty, the accumbal extracellular concentration of dopamine was recorded (at 5 min intervals) for a period of 90 min.

Both LR and HR were injected with 1 mg/kg of RES on day 1 and 15 mg/kg of COC on day 2. Because 1 mg/kg of RES had no effect on the COC-induced increase of accumbal dopamine in HR, a new group of HR was pre-treated with 2 mg/kg of RES on day 1. All drugs were given i.p. in a volume of 1 mL/kg. At the end of the microdialysis experiments, rats were given an overdose of pentobarbital and were intracardially perfused with *p*-formaldehyde. Vibratome sections were cut to verify the location of the microdialysis probe.

### Analysis of the data (experiments 1–3)

Data were statistically analyzed using an anova with the factor type of rat (experiments 1 and 2) or the factors type of rat, treatment, and time (experiment 3). In case HR and LR were differentially sensitive to COC, the effects of RES on the effects of COC were statistically analyzed per type of rat. The relationship between the mean COC-induced increase of accumbal extracellular dopamine and the response to novelty on the open-field (traveled distance and habituation time) were evaluated by mean of Pearson's two-tailed correlation analysis. All data were expressed as mean ± SEM. A probability level of *p* < 0.05 was taken as significant in every test.

## Results

### Open-field selection

The open-field selection procedure revealed 24% LR and 30% HR. The average distance traveled in 30 min was 3493 ± 191 and 8643 ± 373 cm in LR and HR, respectively. The average habituation time was 324 ± 28 s in LR and 1340 ± 66 s in HR. Rats that did not fulfill the criteria (46%) were not included in this study.

### Vesicular levels of accumbal dopamine (experiment 1)

Histological verification revealed that three LR and three HR had to be excluded because incorrect placement of the punch needle. Figure 1 shows the coronal region of the nucleus accumbens in which all correctly placed punches were located.

**Fig. 2:**
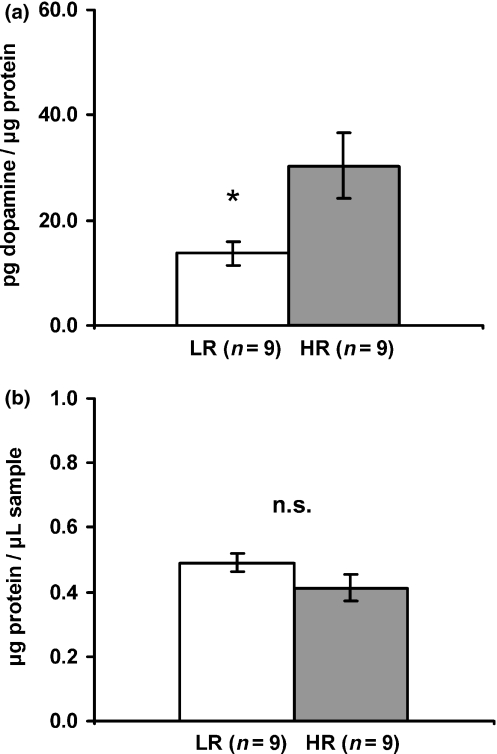
(a) Vesicular levels of accumbal dopamine. The amount of vesicular dopamine (pg) was determined in punches of accumbal tissue. Vesicular dopamine levels were normalized against the protein levels of supernatant 1. *Significant difference between LR (*n* = 9) and HR (*n* = 9) rats (one-way anova). (b) Accumbal protein levels. The total levels of general protein of supernatant 1 did not differ between LR (*n* = 9) and HR (*n* = 9). n.s, no significant differences (one-way anova). All data were expressed as mean ± SEM.

The vesicular levels of accumbal dopamine are depicted in Fig. 2 (LR: *n* = 9 and HR: *n* = 9). The nucleus accumbens of LR was marked by smaller levels of dopamine inside storage vesicles than the nucleus accumbens of HR [Fig. 2a; one-way anova: type effect *F*_(1,16)_ = 6.100, *p* = 0.025]. The accumbal levels of general protein were equal in LR and HR [Fig. 2b; one-way anova: type effect: n.s.], demonstrating that similar pieces of tissue were punched from the nucleus accumbens of both types of rat.

### Accumbal VMAT-2 levels and total levels of accumbal dopamine (experiment 2)

**Fig. 3:**
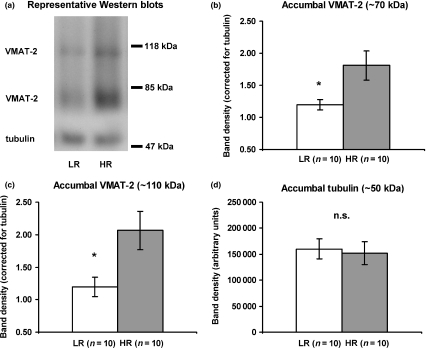
(a) Representative western blots of one LR (left) and one HR (right) using anti β-tubulin and anti vesicular monoamine transporter-2 (VMAT-2) antibodies. Western blot analysis was performed on punches of accumbal tissue. One band for tubulin and two bands for VMAT-2 were observed. (b–c) Immunoreactivity levels of the relatively small accumbal VMAT-2 protein of ∼70 kDa and the relatively large accumbal VMAT-2 protein of ∼110 kDa in LR and HR. VMAT-2 levels were quantified using tubulin for normalization. *Significant differences between LR (*n* = 10) and HR (*n* = 10) rats (one-way anova). (d) Immunoreactivity levels of the accumbal tubulin protein in LR and HR. Tubulin levels did not differ between LR (*n* = 10) and HR (*n* = 10). n.s, no significant differences (one-way anova). All data were expressed as mean ± SEM.

**Fig. 4:**
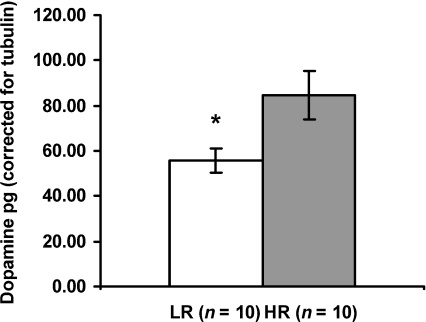
Total levels of accumbal dopamine. The total amount of dopamine (pg) was determined in punches of accumbal tissue. Dopamine levels were quantified using tubulin for normalization (Fig. 3d). *Significant difference between LR (*n* = 10) and HR (*n* = 10) rats (one-way anova). All data were expressed as mean ± SEM.

The accumbal VMAT-2 immunoreactivity levels are depicted in Fig. 3 (LR: *n* = 10 and HR: *n* = 10). The anti-VMAT-2 antibody labeled both a relatively small protein of ∼70 kDa and a relatively large protein of ∼110 kDa (Fig. 3a; Yao *et al.* 2004; Yao and Hersh 2007). A one-way anova revealed that the levels of both the small and the large VMAT-2 protein were significant less in the nucleus accumbens of LR than of HR [Fig. 3b; (VMAT-2 ∼70 kDa): type-effect: *F*_(1,18)_ = 6.490, *p* = 0.020; Fig. 3c (VMAT-2 ∼110 kDa): type effect: *F*_(1,18)_ = 6.822, *p* = 0.018]. The antibody raised against the loading control tubulin selectively labeled a protein of ∼50 kDa (Fig. 3a; Kong *et al.* 1999). The accumbal tubulin levels were equal in LR and HR (Fig. 3d; one-way anova: type effect: n.s.), demonstrating that similar pieces of tissue were punched from the nucleus accumbens of both types of rat (Hedtjarn *et al.* 2002). The total levels of accumbal dopamine are depicted in Fig. 4 (LR: *n* = 10 and HR: *n* = 10). The total levels of dopamine were significant less in the nucleus accumbens of LR than in the nucleus accumbens of HR [Fig. 4; one-way anova: type-effect: *F*_(1,18)_ = 6.000, *p* = 0.023].

### Effects of reserpine on the cocaine-induced increase of extracellular accumbal dopamine (experiment 3)

The dialysis probes of the present study were located in the same region of the nucleus accumbens as the dialysis probes of a previous study (see Fig. 2 of Verheij and Cools 2007). Histological verification revealed that two HR and two LR had to be excluded because of incorrect placement of the dialysis probe. One additional HR had to be excluded from analysis because of obstruction of the microdialysis probe.

The baseline absolute concentration of extracellular accumbal dopamine was 0.68 ± 0.11 pg/10 μL in LR (mean ± SEM of rats belonging to the pooled groups of solvent-treated LR: *n* = 8 + 8 = 16) and 0.84 ± 0.15 pg/10 μL in HR (mean ± SEM of rats belonging to the pooled groups of solvent-treated HR: *n* = 9 + 8 = 17). Extracellular dopamine levels after RES were 0.58 ± 0.07 pg/10 μL in LR (1 mg/kg: *n* = 8) and 0.31 ± 0.05 pg/10 μL in HR (1 and 2 mg/kg pooled: *n* = 8 + 9 = 17). The RES-induced decrease of the basal levels of dopamine in LR [100%– (0.58/0.68 pg) = 15%] and HR [100%– (0.31/0.84 pg] = 63%) are very similar to the previously reported RES-induced decrease of the basal levels of dopamine in these rats (Verheij and Cools 2007). No rat had to be excluded because of undetectable dopamine levels.

**Fig. 5:**
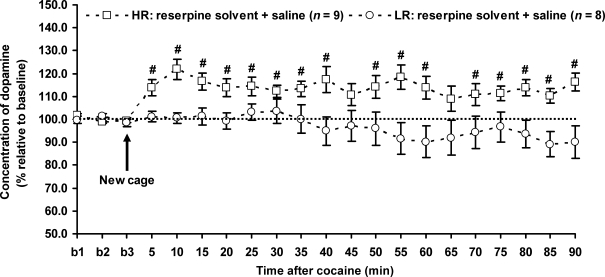
Effects of novelty (new cage) on the extracellular levels of dopamine in the nucleus accumbens in reserpine-solvent and saline-treated LR (circles) and reserpine-solvent and saline-treated HR (squares). Reserpine-solvent (1 mL/kg, i.p.) was administered 24 h before saline (1 mL/kg, i.p.). Rats (LR: *n* = 8 and HR: *n* = 9) were exposed to novelty immediately after the saline injection. Accumbal dopamine levels after novelty are expressed as percentage of baseline accumbal dopamine levels. The horizontal line represents basal dopamine levels (=100%). ^#^Significant increase relative to baseline (one sample *t*-test). All data were expressed as mean ± SEM.

The effects of saline (=solvent of COC) on the extracellular amount of accumbal dopamine in novelty-challenged LR and HR are depicted in Fig. 5 (LR saline: *n* = 8 and HR saline: *n* = 9). The accumbal dopamine response to novelty was larger in saline-treated HR than in saline-treated LR [Fig. 5; two-way anova: type × time effect: n.s; type effect: *F*_(1,15)_ = 12.733, *p* = 0.003]. One sample *t*-test revealed that the extracellular levels of dopamine significantly increased from baseline at 16 out of the 18 time points in control HR, whereas the extracellular levels of dopamine did not differ from baseline at any time point in control LR (Fig. 5).

**Fig. 6:**
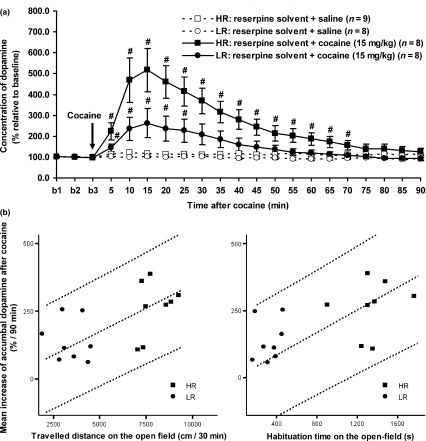
(a) Effects of 15 mg/kg of cocaine on the extracellular levels of dopamine in the nucleus accumbens of novelty-challenged LR (circles) and novelty-challenged HR (squares). Accumbal dopamine levels after cocaine (filled, LR: *n* = 8 and HR: *n* = 8) or saline (open, LR: *n* = 8 and HR: *n* = 9) are expressed as percentage of baseline accumbal dopamine levels. ^#^Significant increase relative to saline (Student's *t*-test). All data were expressed as mean ± SEM. (b) Correlation of traveled distance (left)/habituation time (right) on the open-field and accumbal dopamine increase after cocaine. Traveled distance is expressed as locomotor activity (cm) during 30 min on the open-field. Habituation time is expressed as the duration of the period (s) that started as soon as the rat began to explore the open-field and ended as soon as the locomotor activity stopped for at least 90 s. Accumbal dopamine levels are expressed as mean increase from baseline during 90 min after cocaine [LR (circles): *n* = 8 and HR (squares): *n* = 8]. The dotted lines represent the regression based fit line ± the prediction interval at a confidence level of 95%. One single dot represents one single rat.

The effects of 15 mg/kg of COC on the extracellular amount of accumbal dopamine in novelty-challenged rats are depicted in Fig. 6a (LR COC: *n* = 8 and HR COC: *n* = 8]. COC increased the extracellular dopamine levels in both HR [Fig. 6a; two-way anova: treat × time effect: *F*_(18,270)_ = 11.832, *p* < 0.001] and LR [Fig. 6a; two-way anova: treat × time effect: *F*_(18,252)_ = 5.163, *p* < 0.001]. However, the COC-induced increase of dopamine was stronger in HR than in LR [Fig. 6a; three-way anova: type × treat × time effect: *F*_(18,522)_ = 2.889, *p* < 0.001]. Accumbal dopamine levels increased during the first 70 min in COC-treated HR (Fig. 6a; Student's *t*-test), whereas accumbal dopamine levels increased only during the first 25 min in COC-treated LR (Fig. 6a; Student's *t*-test). Pearson's analysis revealed that both traveled distance and habituation time on the open-field positively correlated with the mean COC-induced increase of accumbal extracellular dopamine [Fig. 6b; LR and HR pooled (*n* = 16): distance (left): *R* = 0.553, *p* = 0.02; habituation time (right): *R* = 0.572, *p* = 0.02].

**Fig. 7:**
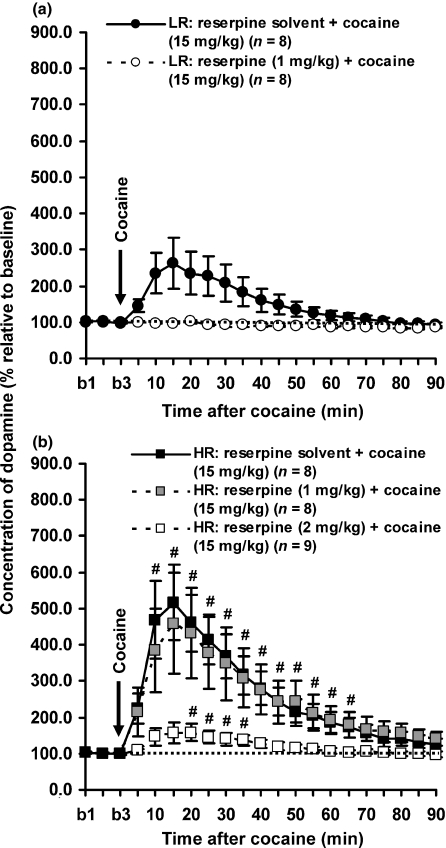
Effects of 1 and 2 mg/kg of reserpine (RES) on the cocaine-induced increase of accumbal extracellular dopamine levels in novelty-challenged LR (a) and novelty-challenged HR (b). Cocaine-induced accumbal dopamine levels in rats treated with solvent (LR: *n* = 8 and HR: *n* = 8) or RES (1 mg/kg: LR: *n* = 8 and HR: *n* = 8; 2 mg/kg: HR: *n* = 9) are expressed as percentage of baseline accumbal dopamine levels. The horizontal line represents basal dopamine levels (=100%). RES reduced baseline levels of dopamine in LR [solvent vs. RES (1 mg/kg): reduction: 100%– (0.58/0.68 pg) = 15%] and HR [solvent vs. RES (1 and 2 mg/kg pooled): reduction: 100%– (0.31/0.84 pg) = 63%]. ^#^Significant dopamine increase after RES (one sample *t*-test). All data were expressed as mean ± SEM.

The effects of 1 mg/kg of RES on the COC-induced increase of extracellular accumbal dopamine are depicted in Fig. 7 (LR: RES 1 mg/kg + COC: *n* = 8 and HR: RES 1 mg/kg + COC: *n* = 8). The dose of 1 mg/kg of RES strongly reduced the COC-induced increase of extracellular dopamine in LR [Fig. 7a; two-way anova: treat × time effect: *F*_(18,252)_ = 5.263, *p* < 0.001]. In fact, one sample *t*-test revealed that accumbal dopamine did not anymore increase from baseline at any time point in these rats (Fig. 7a). The dose of 1 mg/kg of RES did not at all affect the COC-induced increase of extracellular dopamine levels in HR [Fig. 7b; two-way anova: treat × time effect: n.s; treat effect: n.s.]. One sample *t*-test revealed that accumbal dopamine significantly increased from baseline in HR during the first 65 min (Fig. 7b).

The effects of 2 mg/kg of RES on the extracellular amount of accumbal dopamine in COC-treated HR are also depicted in Fig. 7 (HR: RES 2 mg/kg + COC: *n* = 9). A two-way anova revealed that the effects of RES were dose-dependent [Fig. 7b; dose × time effect: *F*_(36,396)_ = 3.135, *p* < 0.001]. The dose of 2 mg/kg of RES strongly reduced the COC-induced increase of accumbal dopamine in HR [Fig. 7b; two-way anova: treat × time effect: *F*_(18,270)_ = 8.272, *p* < 0.001]. One sample *t*-test revealed that dopamine significantly increased from baseline only during the first 35 min [Fig. 7b].

## Discussion

### Accumbal levels of vesicular dopamine and VMAT-2 (experiments 1 + 2)

Low responders to novelty displayed smaller amounts of dopamine inside the storage vesicles of the nucleus accumbens than HR (see Fig. 2). These results can be explained by the finding that LR had less accumbal VMAT-2 than HR (see Fig. 3). It must be noted that two VMAT-2 proteins of different molecular size were identified. It has previously been shown that VMAT-2 proteins are expressed in two morphological distinct types of storage vesicles (Nirenberg *et al.* 1995, 1996, 1997). The small molecular size of VMAT-2 (∼70 kDa) has been found to be localized on small synaptic vesicles, whereas the large molecular size of VMAT-2 (∼110 kDa) has been found to be localized on large dense core vesicles, depending on the glycosylation of the protein (Yao *et al.* 2004; Yao and Hersh 2007). The present finding that LR displayed lower levels of both types of VMAT-2 than HR, indicates that LR are marked by smaller amounts of accumbal dopamine in both small and large vesicles than HR. These smaller levels of vesicular dopamine in LR than in HR may well account for the finding that the total levels of dopamine were smaller in LR than in HR (see Fig. 4).

### Effects of reserpine on cocaine-induced accumbal dopamine levels (experiment 3)

Cocaine increased the extracellular accumbal dopamine levels more strongly in HR than in LR (see Fig. 6a). These results in novelty-challenged rats are very similar to the previous reported results in non-novelty-challenged rats (Hooks *et al.* 1991b; Chefer *et al.* 2003). In fact, the dopamine increase after novelty hardly contributed to the dopamine increase after COC (see Fig. 6a). It was also demonstrated that both behavioral criteria to select HR and LR on the open-field (traveled distance and habituation time) positively correlated with the COC-induced increase of accumbal dopamine (see Fig. 6b). These data were in agreement with the previously reported notion that the response to novelty can predict the individual-specific response to drugs of abuse (Piazza *et al.* 1989, 1991a; Hooks *et al.* 1991a,b; Cools and Gingras 1998). The relatively low dose of 1 mg/kg of RES reduced the COC-induced increase of extracellular accumbal dopamine in LR, but not in HR (see Fig. 7). A higher dose of 2 mg/kg of RES was required to inhibit the COC-induced increase of accumbal dopamine in HR (see Fig. 7). These data confirm the hypothesis that COC-treated LR are more vulnerable to the RES-induced dopamine depletion than COC-treated HR.

Noradrenaline and serotonin are both known to control the release of dopamine (Kilpatrick *et al.* 1996; Cools and Tuinstra 2003). Because RES ultimately depletes dopamine, noradrenaline, and serotonin, the observed effects of RES may be the result of drug-induced changes in the levels of each of these neurotransmitters. However, the fact that the vesicular levels of dopamine differed between LR and HR, suggests that the observed individual differences in the effects of RES are, most likely, because of individual differences in the RES-induced decrease of the levels of dopamine inside storage vesicles.

One could argue that RES reduced the dopamine response to COC for the reason that RES diminished the basal levels of dopamine. Under the condition that COC blocks the re-uptake of neurotransmitters, low basal levels of extracellular dopamine result in a reduced COC-induced increase of dopamine. This explanation, however, is not supported by the present data. In HR, in which 1 mg/kg of RES strongly reduced the basal levels of dopamine (reduction 63%), the dopamine increase following COC was not inhibited at all (Fig. 7). Moreover, in LR, in which 1 mg/kg of RES only slightly reduced the basal levels of dopamine (reduction 15%), the dopamine increase following COC was completely inhibited (Fig. 7). The fact that the results in RES-treated rats cannot exclusively be explained by the generally accepted mode of action that COC inhibits dopamine re-uptake implies that COC must have an additional mode of action. The finding that RES reduced the extracellular dopamine increase to COC (Fig. 7) indicates that COC facilitates the release of dopamine that is derived from storage vesicles (Sulzer and Rayport 1990).

### Dopamine-releasing action of cocaine

The present data were in agreement with previously reported data demonstrating that the COC-induced release of dopamine is caused by exocytosis (Carboni *et al.* 1989; Yan 2003; Venton *et al.* 2006) and not by the reversal of plasmalemmal transporters (Fischer and Cho 1979; Butcher *et al.* 1988; Sulzer *et al.* 1993; Pifl *et al.* 1995; Scarponi *et al.* 1999). The exact mechanism of action for COC to release dopamine from vesicles is currently unknown. Superfusion studies have demonstrated that the dopamine and noradrenaline-releasing action of COC depends not only on storage pools, but also requires the inhibition of plasmalemmal transporters (Pifl *et al.* 1995, 1999). These data indicate that the COC-induced inhibition of dopamine transporters (DATs), somehow, promotes the dopamine release from vesicles. The notion that both DATs and dopaminergic storage vesicles are involved in the effects of COC in animals is confirmed by the outcome of studies in humans demonstrating that the chronic use of COC produces changes not only in DAT-binding, but also in VMAT-binding (Wilson *et al.* 1996; Little *et al.* 1999, 2003).

A dopamine-releasing action of COC involves that dopaminergic storage vesicles become empty after this drug (Pothos and Sulzer 1998; Pothos 2002). Under the condition that COC depletes dopaminergic storage vesicles, replenishment of these vesicles is required. In fact, it has recently been demonstrated that stimulation of D_1_ and D_2_ receptors promotes the refill of vesicles after dopamine has been released by transporter blockers like COC (Brown *et al.* 2001a,b; Sandoval *et al.* 2002). Given the observed individual differences in COC-induced dopamine release, this D_1_/D_2_-receptor mediated refill of vesicles is expected to be larger in COC-treated HR than in COC-treated LR. This nicely fits in with the finding that HR express more VMAT than LR.

### Effects of novelty on accumbal dopamine levels

The finding that HR that were exposed to saline and novelty were marked by a larger increase of extracellular accumbal dopamine than LR that were exposed to saline and novelty (see Fig. 5) fits in with the available literature reporting that challenged HR are marked by a larger accumbal dopamine response than challenged LR (Piazza *et al.* 1991b; Rouge-Pont *et al.* 1993; Saigusa *et al.* 1999; Verheij and Cools 2007). The finding, however, that the extracellular levels of dopamine did not at all increase in novelty-challenged LR is not in agreement with a previous study (Verheij and Cools 2007). It is important to note that the saline-treated and novelty-challenged rats of the present study were also treated with RES-solvent (see Experimental procedures). It has been shown that repeated exposure to the same stressor reduces, or even prevents, the stress-induced increase of dopamine in the nucleus accumbens (Imperato *et al.* 1992, 1993; Cabib and Puglisi-Allegra 1996a,b). Accordingly, the most likely explanation for the present finding that accumbal dopamine levels did not increase in novelty-challenged LR is that the rats of the present study were stressed twice by a systemic injection (RES solvent on day 1 and saline on day 2), whereas the rats of the previous study were stressed only once (RES solvent on day 1 and no saline on day 2). The previously reported finding that RES blocked the accumbal dopamine increase in novelty-challenged LR (Verheij and Cools 2007), suggest that the long-term processes that are triggered by multiple exposure to stressors (anticipation/adaptation) might be related to dopamine stored in RES-sensitive vesicles. The fact that RES did not at all inhibit the accumbal dopamine increase in novelty-challenged HR (Verheij and Cools 2007) may explain why the dopamine decreasing effects of repeated exposure to injection stress did not occur in these rats.

It has previously been reported that RES strongly reduces the baseline levels of dopamine in HR, but not LR (Verheij and Cools 2007). As discussed above, RES strongly reduces the dopamine response to novelty in LR, but not HR. The present study shows that RES decreased the dopamine response to COC in both types of rat. It is, therefore, suggested that the individual differences in (i) the basal dopamine response, (ii) the dopamine response to novelty, and (iii) the dopamine response to COC are regulated by three distinct neuronal substrates (for details see Verheij and Cools 2008).

## Conclusion

The results of the present study indicate that the search for individual differences in the susceptibility to COC should focus not only on individual differences in the re-uptake mechanisms of dopamine, but also on individual differences in the capacity to store dopamine inside vesicles. The present data give rise to the conclusion that LR contain less dopamine inside accumbal storage vesicles than HR because the nucleus accumbens of LR display lower levels VMAT-2 than the nucleus accumbens of HR. The fact that LR are marked by a relatively small storage pool containing low amounts of VMAT, whereas HR are marked by a relatively large storage pool containing high amounts of VMAT may well explain why COC-treated LR are more vulnerable to the dopamine depleting effects of RES than COC-treated HR.

Although it is likely that several mechanisms contribute to individual differences in the sensitivity to COC, the results of the present study indicate that HR are more sensitive to COC than LR because COC can release more dopamine from accumbal storage vesicles in HR than in LR. Given that this release of vesicular dopamine may be mediated by dopamine re-uptake transporters, it is hypothesized that the individual differences in the COC-induced dopamine increase in HR and LR are due to a combination of individual differences in both dopamine re-uptake (Chefer *et al.* 2003) and vesicular dopamine release (present study).

## Impact

The present findings open the intriguing possibility that drugs that deplete dopaminergic storage vesicles of the mesolimbic system (e.g. RES, Ro 4-1284, and tetrabenazine) might become the drugs of choice for the treatment of COC abuse. Interestingly, recent clinical screening trials on the effects of RES in COC-addicted subjects have already revealed promising results in this respect (Gorelick *et al.* 2004; Berger *et al.* 2005). RES-like agents have also been found to be effective in the treatment of hyperkinetic movements disorders like Huntington's chorea (Huntington study group 2006; Kenney and Jankovic 2006). These results suggest that hyperkinesia may, at least in part, be mediated by dopamine derived from storage vesicles.

In addition to the effects of COC, the effects of methylphenidate (Ritalin) are also known to depend on RES-sensitive storage pools (Scheel-Kruger 1971; Chiueh and Moore 1975; Braestrup 1977; McMillen *et al.* 1980; McMillen 1983; Butcher *et al.* 1991). Methylphenidate is used to treat patients suffering from attention deficit hyperactivity disorder (ADHD). Studies in ADHD patients have revealed large individual differences in the clinical response to methylphenidate (Volkow *et al.* 2002; Volkow and Swanson 2003). Given that HR and LR differ in the size of the storage pools that are affected by these drugs, HR and LR may well be a used as an animal model to study the individual-specific variability in the treatment of ADHD (Wooters *et al.* 2006).

Finally, vesicular uptake is suggested to protect a neuron against the toxic effects of high levels of cytoplasmatic dopamine (Truong *et al.* 2003, 2004). In this respect, it is important to note that HR, which are marked by a large number of VMAT, are less sensitive to the neurotoxic effects of 6-hydroxydopamine than LR, which are marked by a small number of VMAT (van Oosten and Cools 2002).

## Abbreviations used

ADHD: attention deficit hyperactivity disorder
COC: cocaine
DAT: dopamine transporter
HR: high responders to novelty
LR: low responders to novelty
RES: reserpine
VMAT-2: vesicular monoamine transporters-2

## Supplementary material

The following supplementary material is available for this article online:

Appendix S1 Full version of the section experimental procedures.

Fig S1 (A) Representative placement of 3 unilateral microdialysis probe tracks located in the right nucleus accumbens. This material is available as part of the online article from http://www.blackwell-synergy.com.
