# Supplementary material for: Rats that differentially respond to cocaine differ in their dopaminergic storage capacity of the nucleus accumbens
Source: J Neurochem. 2008 Jun;105(6):2122–33. doi: 10.1111/j.1471-4159.2008.05323.x (PMC2492658; doi:10.1111/j.1471-4159.2008.05323.x)
Supplement: Appendix S1 — Full version of the section experimental procedures. [file jnc0105-2122-SD1.doc]

Supplementary experimental procedures

Full version of the section experimental procedures (Verheij et al. 2008). 

Animal care: All experiments were performed in accordance with institutional, national and international guidelines for animal care and welfare. 

Subjects: Adult male LR and HR (LR: n=48, HR: n=59, weight=180-220 g) that were selected from the outbred strain of Nijmegen Wistar rats, were used throughout the study. All rats were reared and housed in macrolon cages (42 x 26 x 15 cm; n=3-4 per cage) under a fixed 12/12 h light/dark cycle (lights on: 07.00 a.m.) in a temperature-controlled room (21 ± 1.7 oC). Water and food pellets were available ad libitum. 

Open-field selection: Rats were individually housed 3 days before the open-field selection procedure (Saigusa et al. 1999; Verheij and Cools 2007). Testing took place between 09.00 h and 17.00 h in a room illuminated by white light of 170 Lux. The rat was placed on a black, square table (160 x 160 cm) made of Perspex. This open-field is 95 cm elevated above the floor and surrounded by a white neutral background (270 x 270 x 270 cm). As described by Cools et al. (1990), behavior was recorded with a computerized automated tracking system for a period of 30 min. Both ambulation and habituation time were used to select LR and HR. Ambulation was defined as the overall distance (cm) traveled in 30 min. Habituation time was defined as the duration of the period (s) that started as soon as the rat began to explore the open-field and ended as soon as the locomotor activity stopped for at least 90 s. Rats that habituated in less than 480 s and walked less than 4800 cm in 30 min were labeled LR, whereas rats that habituated after 840 s and walked more than 6000 cm in 30 min were labeled HR. Habituation time in addition to ambulation was used as selection criterion, because traveled distance per se is not always a reliable criterion (Cools et al. 1997; Saigusa et al. 1999). Directly after the open-field selection, LR and HR were assigned to one of the treatment groups.   

Vesicular levels of accumbal dopamine (Experiment 1) 
 
Accumbal punches: Seven days after the open-field selection, a group of 10 LR and 10 HR were sacrificed by decapitation. Their brains were quickly removed, immediately placed in a stainless steel brain matrix (Electron Microscopy Sciences: Hatfield, USA), frozen on dry ice and subsequently sectioned at 3.0 mm intervals (Mong et al. 2003). Coronal incisions were made at anterior/posterior 9.0 and 12.0 mm (Paxinos and Watson, 1986). The most caudal incision was made at the position where the two optic nerves fuse with the optic chiasm (fig. 5 of main document). From the identified slice, one punch of accumbal tissue was obtained from either side of the brain using a 1.22 mm i.d. stainless steel needle (Szczypka et al. 2001). The anterior commissure was used as a landmark to reliable punch out accumbal tissue (see Fig. 5 of main document). For each rat, tissue of the left and right punch was pooled (total volume: 2 x 3.5 mm3 = 7.0 mm3) and stored at -70 oC until vesicular dopamine levels were measured (see below). The remaining tissue was fixated in 4% paraformaldehyde solution in order to allow histological verification of the exact position of the punch needle. 

Vesicular dopamine: Purified accumbal vesicles were prepared as described by Staal et al. (2000). All steps of the isolation procedure were performed at 4 oC. For each rat, tissue of the left and right accumbal punch was pooled and homogenized in 0.32 M sucrose buffer (pH 7.3) containing 1 mM PMSF and 0.1 mg/ml soybean trypsin inhibitor. The homogenate was centrifuged at 2000g for 10 min. The resulting supernatant (S1) was divided over two aliquots. The first aliquot was used as a measure to quantify the total amount of general protein (Bradford assay). The second aliquot was centrifuged at 10000g for 30 min. The resulting synaptosomal pellet (P2) was resuspended by swirling in 0.32 M sucrose buffer. The crude suspension was subjected to osmotic shock by the addition of cold, distilled, deionized water. The osmolarity was restored by the immediate addition of 0.25 M HEPES and 1.0 M potassium tartrate buffer (pH 7.5). The sample was centrifuged at 20000g for 20 min. The resulting supernatant (S3) was centrifuged at 55000g for 60 min. MgSO4 was added to the supernatant (S4) to bring the final magnesium concentration to 0.9 mM. Purified accumbal vesicles were obtained from the pellet (P5) after ultracentrifugation of supernatant (S4) at 100000g for 45 min. 
Vesicular levels of dopamine were obtained according to the procedures described by Sandoval et al. (2003). The vesicular pellet (P5) was sonicated (Branson cell disruptor: Danbury, US) for approximately 5s in cold tissue buffer (0.05 M sodium phosphate, 0.03 M citric acid buffer with 15% methanol (v/v), pH 2.5) and centrifuged for 15 min at 22000g. The final supernatant (S6) was injected into a HPLC-ECD system (see below) for separation and quantification of vesicular dopamine. All centrifugation steps were performed in a Sorvall Micro ultracentrifuge, type RC-M150GX, rotor: S120AT2 (Kendro Laboratory Products, Newton, US). 
Vesicular dopamine levels were normalized for variation in protein loading using the total protein concentration of the first supernatant (Sandoval et al. 2003). 

Accumbal VMAT-2 levels and total levels of accumbal dopamine (Experiment 2)

Western blot analysis: Seven days after the open-field selection, a group of 12 LR and 12 HR were sacrificed by decapitation and accumbal punches were obtained as described above. Accumbal punches were homogenized in phosphate buffered saline (pH 7.3) containing 6 M Urea, 1% SDS, 1% â-mercaptoethanol, 1 mM PMSF and 0.1 mg/ml soybean trypsin inhibitor (Jensen et al. 1998). For each rat, 15 µg of accumbal protein was size fractioned on an 8% SDS-PAGE gel and transferred to nitrocellulose membranes (Protran, Schleicher & Schuell, Keene, US). Following blocking in 5% skimmed milk / 1% Tween-20 / PBS solution for 1 hr at RT, blots were incubated with an anti VMAT-2 (1:1000; AB1767, Chemicon: Hampshire, UK) and an anti -tubulin (1:3000, E7, Chu and Klymkowsky 1989) antibody overnight at 4C. After extensively washing with 1% skimmed milk / 1% Tween-20 / PBS solution, blots were incubated with a peroxidase conjugated secondary antibody (1:5000) for 45 min at RT, and subsequently washed with 1% skimmed milk / 1% Tween-20 / PBS. Proteins on Western blots were immunodetected using Lumi-Light (plus) substrate (Roche Diagnostics, Mannheim, Germany) and subsequently exposed to a bioimaging system. Hybridization signals were analyzed using the Labworks 4.0 program (UVP bioimaging systems, Cambridge, UK). Band intensities were corrected for the background. 

Total dopamine: In addition to the vesicular levels of accumbal dopamine, the total levels of accumbal dopamine were assessed. For quantification of the total levels of accumbal dopamine, the samples that were used to asses the amount of VMAT (see above) were diluted in 0.1 M HCl  (1:100) and immediately injected into the HPLC-ECD system (see below). 
	VMAT and total dopamine levels were normalized for variation in protein loading using the levels of tubulin (Hedtjarn et al. 2002). 

Effects of reserpine on the cocaine-induced increase of extracellular accumbal dopamine (Experiment 3):

Surgery: One day after the open-field selection, a group of 26 LR and 37 HR were unilaterally implanted with stainless steel guide cannulas (length: 5.5 mm, outer diameter: 0.65 mm, inner diameter: 0.3 mm) directed at the right nucleus accumbens according to previously described procedures (Tuinstra and Cools 2000; Verheij and Cools 2007). Under sodium pentobarbital anesthesia (60 mg/kg, volume: 1 ml/kg, i.p.) rats were placed in a stereotaxic apparatus and the following coordinates were used according to the atlas of Paxinos and Watson (1986): anterior: +10.6 mm (relative to the interaural line) and lateral: -1.5 mm (relative to the midline suture). The guide cannula was lowered 5.5 mm relative to the dura surface resulting in a vertical coordinate of +3.5 mm for the cannula tip.  Finally, the cannula was angled 10o laterally to the right side (see Fig. I of supplementary material). Screws and cement were used to fixate the cannula to the skull. The guide cannula contained an inner cannula to prevent infections and occlusions. 
The rats were allowed to recover from surgery for the next 7 to 10 days in Plexiglas dialysis cages (25 x 25 x 35 cm) covered with sawdust on the floor (Saigusa et al. 1999; Verheij and Cools 2007). On 3 consecutive days just prior to the start of the experiment, each rat was gently picked up. This handling procedure was repeated 3 times per day (Saigusa et al. 1999; Verheij and Cools 2007). 

Reserpine treatment: At the first day of the experiment, a dialysis probe (type A-I-8-02, outer diameter: 0.22 mm, 50000-molecular-weight cut-off, Eicom, Tokyo, Japan) was carefully inserted into the brain of a conscious rat. The tip of the dialysis probe protruded 2 mm below the distal end of the guide cannula. The probe had an in vitro recovery of 10-12% for dopamine (Saigusa et al. 1999; Verheij and Cools 2007). 4 Hours following probe insertion, HR and LR were injected with RES (1 or 2 mg/kg: see below) or its solvent (volume: 1 ml/kg, i.p.). Because the RES-induced decrease of the accumbal extracellular dopamine levels observed in HR and LR on day 1 have already been reported (Verheij and Cools 2007), we now report only the effects of RES on the accumbal extracellular dopamine levels of day 2. 

Microdialysis: At the second day of the experiment, the inlet and outlet of the dialysis probe were connected to a swivel and accumbal dialysates were analyzed for dopamine according to previously described procedures (De Leonibus et al. 2006). The probe was perfused at a rate of 2.0 ìl/min with modified Ringer solution and the outflow was collected into a stand-alone HPLC-ECD system (HTEC-500, software version 1.02) of Eicom (Tokyo, Japan). Dopamine was separated from the remaining neurotransmitters by means of reversed phase, ion-paring liquid chromatography using an Eicompak PP-ODS column (particle size: 2 ìm, 4.6 x 30 mm, Eicom, Tokyo, Japan) in combination with a mobile phase containing 1% of methanol (flow rate: 500 µl/min, temp: 25 °C). The concentration of dopamine was measured by setting the working electrode of the electrochemical detector at + 400 mV against a silver/silver-chloride reference electrode. The HPLC-ECD unit was calibrated with a standard dopamine solution twice before each experiment. The detection limit was about 30 fg per sample (= 10 ìl). 
	At 4 h following the start of the microdialysis, the extracellular accumbal concentration of dopamine (pg/sample) is known to reach a stable baseline ± 10% (Saigusa et al. 1999; Tuinstra and Cools 2000; van der Elst et al. 2005; De Leonibus et al. 2006; Verheij and Cools 2007). As soon as 4 h had past and successive samples differed less than 10%, 3 baseline samples were taken. The average of these 3 samples served as control value (100%) to study the drug-induced changes of accumbal dopamine. 

Cocaine treatment: Immediately after the third baseline sample was taken, rats that were treated with RES or its solvent on day 1 were injected with COC (15 mg/kg) or saline (volume 1 ml/kg, i.p.). Because the effects of COC on the accumbal dopamine levels in LR and HR under non-challenged conditions have already been reported (Hooks et al. 1991; Chefer et al. 2003), the present study focused only on the effects of COC on the accumbal dopamine levels in LR and HR under novelty-challenged conditions. Immediately after the injection of COC or saline, rats were exposed to a cage that was slightly larger than the home cage (new dimensions: 30 x 30 x 35 cm) and lacked sawdust on the floor (Verheij and Cools 2007). LR and HR were left undisturbed in their new environment and the accumbal extracellular concentration of dopamine was recorded (at 5 min intervals) for an additional period of 90 min. 

Treatment strategy: COC was given at 24 h after RES because the dopamine depleting effects of RES are known to be maximal at this time (Verheij and Cools 2007).  Both LR and HR were injected with 1 mg/kg of RES. Because 1 mg/kg of RES had no effect on the COC-induced increase of accumbal dopamine in HR, a new group of HR was pre-treated with 2 mg/kg of RES. The relatively low doses of 1 and 2 mg/kg of RES were chosen because it was previously shown that these doses selectively affect RES-sensitive dopaminergic storage vesicles (Cools and Verheij 2002).

Histology: At the end of the microdialysis experiments, rats were given an overdose of sodium-pentobarbital (250 mg/kg, i.p.) and were intracardially perfused with 60 ml 4% paraformaldehyde solution. Vibratome sections (100 ìm) were cut to determine the exact location of the microdialysis probe (see Fig. I of supplementary material).     

Solutions: The following solutions were used: 1) Modified Ringer solution: 147 mM NaCl, 4 mM KCl, 1.1 mM CaCl2.2H2O and 1.1 mM MgCl2.6H2O were dissolved in ultra pure water (pH 7.4), 2) Mobile phase (pH = 6.0): 0.1 M phosphate buffer (NaH2PO4.2H2O : Na2HPO4.12H2O, ratio 25:4),  2.0 mM sodium 1-decanesulphonate and 0.1 mM di-sodium EDTA were dissolved in ultra pure water (>18 MΩ) containing 1% methanol, 3) Dopamine standard solution: 50 pg in 10 µl of 0.1 M HCl solution, 4) Reserpine: ampoules containing 1 mg or 2 mg of RES per ml solvent (Verheij and Cools 2007), 5) Reserpine solvent: 30 mg dl-methionine dissolved in 10 ml aquadest containing 6.75% propylene glycol (Verheij and Cools 2007). The pH of the RES solution and that of its solvent was adjusted to 2.4 using phosphoric acid.

Analysis of the data (experiment 1-3): Data were statistically analyzed using an ANOVA with the factor type of rat (experiment 1 and 2) or the factors type of rat, treatment and time for repeated measures (experiment 3). In case HR and LR were differentially sensitive to COC, the effects of RES on the effects of COC were statistically analyzed per type of rat. One sample t-tests were used to evaluate whether a specific treatment significantly changed accumbal dopamine levels from baseline. The relationship between the mean COC-induced increase of accumbal extracellular dopamine during 90 min and the response to novelty on the open-field (traveled distance and habituation time) were evaluated by means of Pearson's 2-tailed correlation analysis in a pooled group of LR and HR. All data are expressed as mean ± SEM. A probability level of p<0.05 was taken as significant in every test. SPSS for Windows (Release 12.0) was used to statistically analyze the data. 

References

Chefer V. I., Zakharova I. and Shippenberg T. S. (2003) Enhanced responsiveness to novelty and cocaine is associated with decreased basal dopamine uptake and release in the nucleus accumbens: quantitative microdialysis in rats under transient conditions. J. Neurosci. 23, 3076-3084.
Chu D. T. and Klymkowsky M. W. (1989) The appearance of acetylated alpha-tubulin during early development and cellular differentiation in Xenopus. Dev. Biol. 136, 104-117.
Cools A. R., Brachten R., Heeren D., Willemen A. and Ellenbroek B. (1990) Search after neurobiological profile of individual-specific features of Wistar rats. Brain Res. Bull. 24, 49-69.
Cools A. R., Ellenbroek B. A., Gingras M. A., Engbersen A. and Heeren D. (1997) Differences in vulnerability and susceptibility to dexamphetamine in Nijmegen high and low responders to novelty: a dose-effect analysis of spatio-temporal programming of behaviour. Psychopharmacology (Berl) 132, 181-187.
Cools A. R. and Verheij M. M. M. (2002) Alpha-adrenoceptors, but not beta-adrenoceptors, regulate the extracellular release of dopamine stored in reserpine-sensitive vesicular pools in the Nucleus Accumbens. Abstract Viewer and Itinerary Planner 2002. Society for neuroscience 32nd annual meeting, Orlando. Program No. 736.3.2002.
De Leonibus E., Verheij M. M., Mele A. and Cools A. (2006) Distinct kinds of novelty processing differentially increase extracellular dopamine in different brain regions. Eur. J. Neurosci. 23, 1332-1340.
Hedtjarn M., Leverin A. L., Eriksson K., Blomgren K., Mallard C. and Hagberg H. (2002) Interleukin-18 involvement in hypoxic-ischemic brain injury. J. Neurosci. 22, 5910-5919.
Hooks M. S., Jones G. H., Smith A. D., Neill D. B. and Justice J.-B. J. (1991) Response to novelty predicts the locomotor and nucleus accumbens dopamine response to cocaine. Synapse 9, 121-128.
Jensen P. H., Nielsen M. S., Jakes R., Dotti C. G. and Goedert M. (1998) Binding of alpha-synuclein to brain vesicles is abolished by familial Parkinson's disease mutation. J. Biol. Chem. 273, 26292-26294.
Mong J. A., Devidze N., Frail D. E., O'Connor L. T., Samuel M., Choleris E., Ogawa S. and Pfaff D. W. (2003) Estradiol differentially regulates lipocalin-type prostaglandin D synthase transcript levels in the rodent brain: Evidence from high-density oligonucleotide arrays and in situ hybridization. Proc. Natl. Acad. Sci. USA 100, 318-323.
Paxinos G. and Watson C. (1986) The Rat Brain in Stereotaxic Coordinates. Elsevier Academic Press, San Diego.
Saigusa T., Tuinstra T., Koshikawa N. and Cools A. R. (1999) High and low responders to novelty: effects of a catecholamine synthesis inhibitor on novelty-induced changes in behaviour and release of accumbal dopamine. Neuroscience 88, 1153-1163.
Sandoval V., Riddle E. L., Hanson G. R. and Fleckenstein A. E. (2003) Methylphenidate alters vesicular monoamine transport and prevents methamphetamine-induced dopaminergic deficits. J. Pharmacol. Exp. Ther. 304, 1181-1187.
Staal R. G., Hogan K. A., Liang C. L., German D. C. and Sonsalla P. K. (2000) In vitro studies of striatal vesicles containing the vesicular monoamine transporter (VMAT2): rat versus mouse differences in sequestration of 1-methyl-4-phenylpyridinium. J. Pharmacol. Exp. Ther. 293, 329-335.
Szczypka M. S., Kwok K., Brot M. D., Marck B. T., Matsumoto A. M., Donahue B. A. and Palmiter R. D. (2001) Dopamine production in the caudate putamen restores feeding in dopamine-deficient mice. Neuron 30, 819-828.
Tuinstra T. and Cools A. R. (2000) Newly synthesized dopamine in the nucleus accumbens is regulated by beta-adrenergic, but not alpha-adrenergic, receptors. Neuroscience 98, 743-747.
van der Elst M. C., Verheij M. M., Roubos E. W., Ellenbroek B. A., Veening J. G. and Cools A. R. (2005) A single exposure to novelty differentially affects the accumbal dopaminergic system of apomorphine-susceptible and apomorphine-unsusceptible rats. Life Sci. 76, 1391-1406.
Verheij M. M. and Cools A. R. (2007) Differential contribution of storage pools to the extracellular amount of accumbal dopamine in high and low responders to novelty: effects of reserpine. J. Neurochem. 100, 810-821.

Figure legends

Fig I (A) Representative placement of 3 unilateral microdialysis probe tracks located in the right nucleus accumbens. The probe protrudes 2 mm below the distal end of the guide cannula. (B-D) Schematic illustration of coronal brain sections containing the nucleus accumbens. The brain region in which correctly placed probes were found is indicated in gray. IA corresponds to the distance (mm) from the interaural line according to Paxinos and Watson (1986), NAC=nucleus accumbens, CPU=caudate putamen, CC=corpus callosum.
