# Supplementary figures and images for: Rats that differentially respond to cocaine differ in their dopaminergic storage capacity of the nucleus accumbens
Source: J Neurochem. 2008 Jun;105(6):2122–33. doi: 10.1111/j.1471-4159.2008.05323.x (PMC2492658; doi:10.1111/j.1471-4159.2008.05323.x)

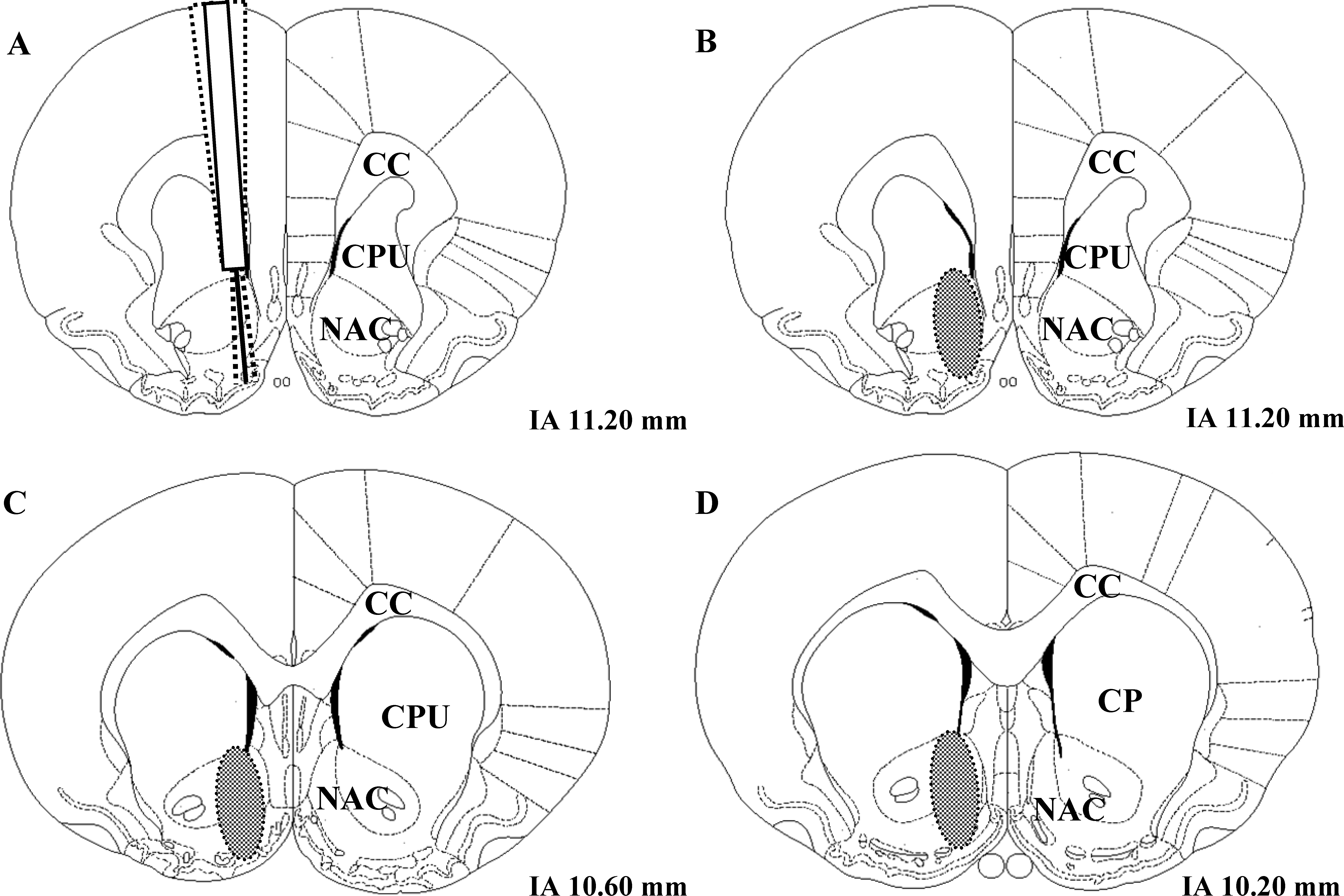

Supplement: Fig S1 — (A) Representative placement of 3 unilateral microdialysis probe tracks located in the right nucleus accumbens. This material is available as part of the online article from http://www.blackwell-synergy.com. [file jnc0105-2122-SD2.tif]
